# Age, skin color, self-rated health, and depression associated with co-occurrence of obesogenic behaviors in university students: a cross-sectional study

**DOI:** 10.1590/1516-3180.2022.0301.R1.10102022

**Published:** 2022-12-19

**Authors:** Bruna Carolina Rafael Barbosa, Magda do Carmo Parajára, Waléria de Paula, Elaine Leandro Machado, Adriana Lúcia Meireles

**Affiliations:** I MSc. Doctoral Student, Postgraduate Program in Health and Nutrition, School of Nutrition, Universidade Federal de Ouro Preto (UFOP), Ouro Preto (MG), Brazil.; II MSc. Doctoral Student, Postgraduate Program in Health and Nutrition, School of Nutrition, Universidade Federal de Ouro Preto (UFOP), Ouro Preto (MG), Brazil.; III MSc. Doctoral Student, Postgraduate Program on Pharmaceutical Sciences, School of Pharmacy, Universidade Federal de Ouro Preto (UFOP), Ouro Preto (MG), Brazil.; IV PhD. Professor, Department of Preventive and Social Medicine, Faculty of Medicine, Universidade Federal de Minas Gerais (UFMG), Belo Horizonte (MG), Brazil.; V PhD. Professor, Department of Clinical and Social Nutrition, School of Nutrition, Universidade Federal de Ouro Preto (UFOP), Ouro Preto (MG), Brazil.

**Keywords:** Obesity, Health risk behaviors, Students, Risk factors, Cross-sectional studies, Obesogenic behavior, University students, Clustering

## Abstract

**BACKGROUND:**

The university context plays an important role in the health-disease process since students are potentially vulnerable to obesogenic behaviors that can influence long-term health.

**OBJECTIVE:**

To estimate the prevalence of and factors associated with the co-occurrence of obesogenic behaviors among university students.

**DESIGN AND SETTING:**

This was a cross-sectional study at a Brazilian public university.

**METHODS:**

This study was conducted with all university students in the first and second semesters of 2019 at Universidade Federal de Ouro Preto, Minas Gerais, Brazil. Data were collected between April and September 2019, using a self-administered questionnaire. The outcome was the co-occurrence of obesogenic behaviors, measured as the sum of three risk behaviors: inadequate eating practices, leisure-time physical inactivity, and sedentary behavior. A Venn diagram was used to evaluate the simultaneous occurrence of risk behaviors. Pearson’s chi-square test and multivariate logistic regression were used for statistical analyses.

**RESULTS:**

A total of 351 students participated in the study. Inadequate eating practices constituted the most prevalent isolated risk behavior (80.6%), which was also the most prevalent when combined with sedentary behavior (23.6%). University students aged 20 years or younger, with non-white skin color, poor self-rated health, and symptoms of depression had increased chances of simultaneous occurrence of obesogenic behaviors.

**CONCLUSION:**

These findings highlight the importance of developing and implementing actions to reduce combined obesogenic behaviors in the university environment. Institutions should focus on creating an environment that promotes health-protective behaviors such as physical activity and healthy eating.

## INTRODUCTION

The prevalence of obesity has increased rapidly in recent decades in both developed and developing countries, reaching the status of a global pandemic. This condition is characterized as both a disease and a risk factor for other chronic non-communicable diseases (NCDs). The etiology of this condition is multifactorial, resulting from a complex interaction between genetic, individual, sociocultural, economic, environmental, and behavioral factors. Epidemiological studies have demonstrated the relationship between important risk factors associated with this morbidity, which represents one of the biggest public health problems today.^1-5^

Many health risk behaviors, such as inadequate eating practices, low levels of physical activity, and sedentary behavior (SB), are considered independent risk factors for being overweight, contributing to increased morbidity and mortality due to NCDs.^6-8^ However, exposure to these risk factors does not occur in an isolated manner^9^ but in a group or simultaneously, increasing the risk of becoming overweight and obese.^10,11^

Although studies have evaluated the aggregation of multiple health risk behaviors, especially in the general adult population,^12^ few studies have focused on university students.^13,14^ University enrollment represents a period of health risk for young adults, as it results in numerous changes in the student’s life, including increased opportunities to initiate and establish unhealthy behaviors that favor weight gain.^15^ Additionally, it is observed that other factors are associated with obesogenic behaviors among university students during academic life, with emphasis on those related to sociodemographic, individual, social, and environmental characteristics.^16^

Understanding potentially obesogenic behavioral risk factors among university students is imperative for identifying more susceptible groups and recognizing the health effects of these factors, to facilitate the development of prevention and health promotion strategies targeted at the university environment. Additionally, this information can contribute to more effective public policies to reduce the rates of obesity- and overweight-related NCDs.

## OBJECTIVE

This study therefore aimed to estimate the prevalence of co-occurrence of obesogenic behaviors and their associated factors in university students.

## METHODS

### Study design and population

This cross-sectional study was integrated with a project on anxiety and depression among university students titled “Symptoms of Anxiety and Depression among University Students of Minas Gerais: a longitudinal study” (Projeto sobre Ansiedade e Depressão em Universitários - PADu). This study was approved by the Research Ethics Committee of the Universidade Federal de Ouro Preto (UFOP) under CAAE no. 19467919.5.0000.5150 on December 19, 2019.

PADu is a longitudinal study conducted with university students entering undergraduate courses offered at the campi of Ouro Preto and Mariana of the UFOP. Data will be collected at three different time points (T0—in the first semester of the undergraduate course; T1—after attending two years; T2—after attending four years) to verify behavioral changes during academic life. For the present study, data from the baseline (T0) were used.

The study population included all university students entering the first and second semesters in the 2019 undergraduate courses in architecture and urbanism, performing arts, law, physical education, civil engineering, production engineering, geological engineering, pharmacy, history, journalism, mathematics, medicine, nutrition, and pedagogy. The students’ lists were made available through the UFOP’s teaching sections.

Students who met the following inclusion criteria participated in the research: regularly enrolled in the first period of the undergraduate courses evaluated in the study and aged 18 years or older.

The PADu sample comprised 355 university students. However, the final sample of this study consisted of 351 university students, since four participants did not answer all the questions related to the co-occurrence of obesogenic behaviors.

### Data collection

Data were collected between April and September 2019 by project members who were previously trained to apply the instrument and clarify possible doubts of the students. A pilot study was conducted with students attending the eighth period of the nutrition course in the second semester of 2018 who would therefore not participate in the sample.

The questionnaires were administered during class hours, after taking prior appointments, and the teacher’s presence in each selected course. The researchers oriented the university students about the study, risks, and benefits. They were also informed that their participation was voluntary and anonymous. Those who agreed to participate signed the informed consent form and answered a questionnaire consisting of socioeconomic characteristics, lifestyle habits, and health conditions.

### Variables of the study

The outcome variable (co-occurrence of obesogenic behaviors) was obtained from the sum of three risk behaviors: inadequate eating practices, leisure-time physical inactivity, and SB. The responses were categorized as none to three obesogenic behaviors. These behaviors are justified because they are considered health risk factors and are associated with the most significant burden of NCDs and mortality.^17^

The variable “inadequate eating practices” was obtained through a scale developed and validated by Gabe and Jaime for adults, which measures adherence to healthy eating practices based on the recommendations of the second edition of the Food Guide for the Brazilian Population.^18,19^ For classification purposes, the cut-off points proposed by Gabe and Jaime were used, wherein eating practices were classified as “inadequate” when the sum of the individual scores assigned to the responses for each alternative resulted in a score of up to 31 points, at “risk” when the score was between 32 and 41 points, and “adequate” when it was greater than 41 points.^20,21^ Subsequently, for the present study, eating practices were recategorized as “adequate” and “inadequate.”

Leisure-time physical inactivity was assessed using the study Surveillance System for Risk and Protective Factors for Chronic Diseases by Telephone Survey (VIGITEL), with questions such as: “In the last three months, did you practice any physical exercise or sport? (Do not consider physical therapy).”^22^ Participants who answered “no” were classified as “inactive in leisure time,” and those who answered “yes” were considered “active in leisure time.”

SB was included in the study because a growing number of studies characterize it as a health risk factor, different from and independent of physical inactivity, and associated with the occurrence of adverse health effects, such as metabolic syndrome.^9,23^ SB was determined in the questionnaire using the following question: “In your free time, that is, when you are not studying or working, how much time (in hours) do you dedicate to using the cell phone, television, computer, or tablet?” This question was adapted from two questions from VIGITEL.^22^ For each of the screen types evaluated, eight answers were possible: “I don’t use,” “less than an hour,” “between one to two hours,” “between two to three hours,” “between four to five hours,” “between five to six hours,” and “more than six hours.” For analysis purposes, SB was analyzed as a continuous variable and responses were coded as 0, 0.5, 1.5, 2.5, 3.5, 4.5, 5.5, and 6.5 hours, respectively. Subsequently, the responses of the time spent on each type of screen were summed, and the classification of SB was established according to the median. University students with screen time ≤ 6 h were classified as “non-sedentary,” while those with screen time > 6 h were considered “sedentary.”

The explanatory variables included in this study were grouped into two domains: sociodemographic characteristics and health conditions. The variables assessed in the sociodemographic domain included sex (male and female), age (≤ 20 years and > 20 years), skin color (white and non-white—yellow, brown, mulatto, or black), sexual orientation (heterosexual and others—homosexual, bisexual, or asexual), marital status (single and others—married, stable union, widowed, or divorced), and total monthly family income (< three minimum wages and ≥ three minimum wages). The wage value considered in this study refers to the minimum wage in force in Brazil in 2019 (R$ 998.00). The sociodemographic domain comprised the following variables: housing (without and with family members), area of knowledge (life sciences, exact sciences, humanities, and social and applied sciences), and work (no and yes).

In the health condition domain, the following variables were evaluated: self-rated health, categorized as “good” (good and very good) and “bad” (regular, bad, and very bad); anthropometric profile (not overweight or overweight); use of medication for chronic diseases (no and yes); anxiety symptoms (no and yes); depression symptoms (no and yes); and stress symptoms (no and yes).

The anthropometric profile was evaluated by calculating the body mass index (BMI) through the anthropometric measurements of weight and height, self-reported by the participants. The classification was made according to the BMI reference values established by the World Health Organization for adults^24^ and adolescents.^25^ Individuals classified as underweight and eutrophic (BMI < 24.9 kg/m^2^) were grouped in the “not overweight” category and those classified as overweight and obese (BMI ≥ 25 kg/m^2^) in the “overweight” category. Additional details on the anthropometric profile classification methodology can be found in a previous publication.^26^

The variables “anxiety symptoms,” “depression symptoms,” and “stress symptoms” were obtained through the reduced version of the Depression Anxiety Stress Scale-21 (DASS-21).^27^ The scale is composed of a set of three subscales, designed to estimate in a self-reported way the symptoms of anxiety, depression, and stress in the week before data collection. The response scale to the items is a four-point Likert scale ranging from 0 (not applicable) to 3 (applicable most of the time), generating scores that allow the classification of anxiety, depression, and stress symptoms as “normal,” “mild,” “moderate,” “severe,” and “extremely severe.” In the present study, symptoms of mental disorders were re-classified as absence (“no”; normal and mild) and presence (“yes”; moderate, severe, and extremely severe).

### Statistical analysis

The variables were descriptively analyzed using frequency distribution. A Venn diagram was used to represent the simultaneous occurrence of obesogenic behaviors among the evaluated university students. This representation method allows for the comparison and visualization of the overlap and differences among the datasets being analyzed based on the intersections of the graphical shapes.^28,29^

Initially, the chi-square test was performed between the explanatory variables and the co-occurrence of obesogenic behaviors, and those with a P value < 0.20 in the bivariate analysis were included in the multivariate model. Multivariate logistic regression was used to verify the association between the co-occurrence of obesogenic behaviors and explanatory variables. In this analysis phase, three models were structured to represent the co-occurrence of the obesogenic behaviors evaluated: Models 1, 2, and 3 included no behavior versus one behavior, no behavior versus two behaviors, and no behavior versus three behaviors, respectively. For this, we used a reference category for university students with no obesogenic behavior versus the number of obesogenic behaviors (1, 2, or 3). To select sociodemographic and health condition variables, the backward method was adopted, and only the variables that presented a P value of < 0.05 remained in the multivariate model. All the models were adjusted for the variable “sex.” The odds ratio (OR) was used to measure the association with the respective 95% confidence intervals (95% CI). The level of statistical significance was 5%. The analyses were performed using Stata version 13.0 (Stata Corporation, College Station, Texas, United States).

## RESULTS

Of the 351 university students included in this study, 57.6% were female and 65.8% were 20 years or younger, ranging from 18 to 31 years. Most participants self-reported their color or race as white (51.1%), single (95.4%), heterosexual (79.5%), living without family members (66.4%), and not employed (89.2%). Regarding family income, slightly more than half (56.7%) of the students reported a family income higher than or equal to three minimum wages. Regarding the distribution by area of knowledge, 41.0% were from life sciences courses, 34.5% from the humanities and social and applied sciences, and 24.5% from the exact sciences (Table 1).

**Table 1.** Number of obesogenic behaviors in university students entering the Universidade Federal de Ouro Preto in 2019, according to sociodemographic characteristics and health conditions. Ouro Preto, Minas Gerais, 2019 (n = 351)

Variables	n	%	% of obesogenic behaviors				P value*
			0	1	2	3	
**Sex**							**0.013**
Male	149	42.4	8.0	40.3	38.3	13.4	
Female	202	576	10.9	24.7	43.1	21.3	
**Age (n = 348)**							**0.040**
≤ 20 years	229	65.8	6.5	33.6	41.1	18.8	
> 20 years	119	34.2	16.0	27.7	40.3	16.0	
**Skin color (n = 350)**							**0.015**
White	179	51.1	14.5	31.3	36.9	17.3	
Non-white (yellow, brown, mulatto, or black)	171	48.9	4.7	31.0	45.6	18.7	
**Sexual orientation**							0.729
Heterosexual	279	79.5	9.7	32.3	41.2	16.8	
Others (homosexual, bisexual, or asexual)	72	20.5	9.7	27.8	40.3	22.2	
**Marital status**							0.584
Single	335	95.4	9.3	31.6	40.9	18.2	
Others (married, stable union, widowed, or divorced)	16	4.6	18.7	25.0	43.8	12.5	
**Total monthly family income** ^**^							0.077
< 3 minimum wages	152	43.3	5.9	35.5	38.2	20.4	
≥ 3 minimum wages	199	56.7	12.6	28.1	43.2	16.1	
**Housing**							0.091
Without family members	233	66.4	9.9	35.6	38.2	16.3	
With family members	118	33.6	9.3	22.9	46.6	21.2	
**Area of knowledge**							0.365
Life Sciences	144	41.0	11.1	31.2	41.0	16.7	
Exact Sciences	86	24.5	12.8	33.7	40.7	12.8	
Humanities and Social and Applied Sciences	121	34.5	5.8	29.8	41.3	23.1	
**Work**							0.268
No	313	89.2	10.5	32.0	39.6	17.9	
Yes	38	10.8	2.6	26.3	52.7	18.4	
**Self-rated health**							**0.024**
Good (very good and good)	207	59.0	12.6	33.3	40.1	14.0	
Bad (regular, bad, and very bad)	144	41.0	5.6	28.5	42.3	23.6	
**Anthropometric profile (n = 346)**							0.365
Not overweight	269	77.7	10.4	30.9	39.4	19.3	
Overweight	77	22.3	6.5	35.1	45.4	13.0	
**Use of medication for chronic diseases**							0.519
No	303	86.3	9.9	32.7	39.9	17.5	
Yes	48	13.7	8.3	22.9	47.9	20.9	
**Anxiety symptoms (n = 350)**							**< 0.001**
No	201	57.4	11.9	32.8	44.8	10.5	
Yes	149	42.6	6.7	28.9	36.2	28.2	
**Depression symptoms (n = 349)**							**< 0.001**
No	232	66.5	12.1	32.3	44.4	11.2	
Yes	117	33.5	5.1	28.2	35.1	31.6	
**Stress symptoms (n = 349)**							**0.001**
No	222	63.6	13.1	32.4	41.9	12.6	
Yes	127	36.4	3.9	28.3	40.2	27.6	

*P value obtained using Pearson’s Chi-Square test; ^**^The minimum wage in force in Brazil in 2019 = R$ 998.00. In bold: the statistically significant variables in the bivariate analysis.

Regarding health conditions, 41.0% of the university students self-rated their health as bad, 22.3% were overweight, and 13.7% reported using medications for chronic diseases. Anxiety, depression, and stress symptoms were observed to be 42.6%, 33.5%, and 36.4% of the interviewed university students, respectively (Table 1).

Regarding isolation, the most prevalent obesogenic behavior was inadequate eating practices (80.6%; 95% CI: 76.5%–84.8%), followed by SB (49.2%; 95% CI: 44.0–54.5%) and leisure-time physical inactivity (37.3%; 95% CI: 32.2–42.4%).

**Figure 1.**
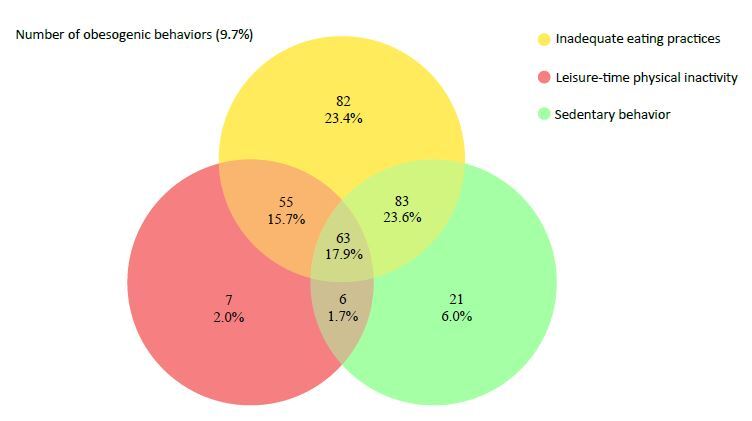
Co-occurrence of obesogenic behaviors (inadequate eating practices, leisure-time physical inactivity, and sedentary behavior) in university students entering the Universidade Federal de Ouro Preto in 2019. Ouro Preto, Minas Gerais, 2019 (n = 351).

Figure 1 shows the co-occurrence of obesogenic behavior. The adoption of inadequate eating practices and SB (23.6%) was observed to be the most prevalent combination of risk behaviors among students, followed by inadequate eating practices, leisure-time physical inactivity, and SB (17.9%), and inadequate eating practices and leisure-time physical inactivity (15.7%). The absence of risk factors was observed in 9.7% of university students.

The prevalence distribution of obesogenic behaviors according to sociodemographic characteristics and health conditions is presented in Table 1. In the bivariate analysis, sex, age, skin color, self-rated health, anxiety, depression, and stress symptoms remained associated with the co-occurrence of obesogenic behaviors among university students.

Table 2 presents the results of the multivariate analysis. In the final adjusted models, the following variables maintained a significant association (P value < 0.05) with the co-occurrence of obesogenic behaviors: age, skin color, self-rated health, and depression symptoms. Age and skin color remained associated, in the three evaluation models.

**Table 2.** Odds ratio (OR) and 95% confidence interval (95% CI) for one or more obesogenic behaviors; multivariate model of sociodemographic characteristics and health conditions associated with the co-occurrence of obesogenic behaviors in university students entering the Universidade Federal de Ouro Preto in 2019. Ouro Preto, Minas Gerais, 2019 (n = 351)

Model one: No obesogenic behavior versus one obesogenic behavior			
Variables	OR	95% CI	P value
**Age (n = 348)**			0.003
> 20 years	1		
≤ 20 years	3.68	(1.58–8.59)	
**Skin color (n = 350)**			0.016
White	1		
Non-white (yellow, brown, mulatto, or black)	3.09	(1.23–7.74)	
**Model two: No obesogenic behavior versus two obesogenic behaviors**			
**Variables**	**OR**	**95% CI**	**P value**
**Age (n = 348)**			0.014
> 20 years	1		
≤ 20 years	2.77	(1.23–6.26)	
**Skin color (n = 350)**			0.001
White	1		
Non-white (yellow, brown, mulatto, or black)	4.61	(1.88–11.31)	
**Self-rated health**			0.033
Good (very good and good)	1		
Bad (regular, bad, and very bad)	2.70	(1.09–6.71)	
**Model three: No obesogenic behavior versus three obesogenic behaviors**			
**Variables**	**OR**	**95% CI**	**P value**
**Age (n = 348)**			0.018
> 20 years	1		
≤ 20 years	3.34	(1.23–9.05)	
**Skin color (n = 350)**			0.027
White	1		
Non-white (yellow, brown, mulatto, or black)	3.31	(1.15–9.58)	
**Depression symptoms**			0.001
No	1		
Yes	6.15	(2.10–18.05)	

OR = odds ratio; CI = confidence interval.

In model 1 (no behavior versus one behavior), individuals aged 20 years or younger [OR: 3.68 (95% CI: 1.58–8.59)] and those who reported colored skin [OR: 3.09 (95% CI: 1.23–7.74)] were more likely to have obesogenic behavior. In model 2 (no behavior versus two behaviors), students who were 20 years or younger [OR: 2.77 (95% CI: 1.23–6.26)], colored skin [OR: 4.61 (95% CI: 1.88–11.31)], and self-rated their health as bad [OR: 2.70 (95% CI: 1.09–6.71)] were more likely to have two obesogenic behaviors simultaneously. In model 3 (no behaviors versus three behaviors), those aged 20 years or younger [OR:3.34 (95% CI: 1.23–9.05)], colored skin [OR: 3.31 (95% CI: 1.15–9.58)], and reported symptoms of depression [OR: 6.15 (95% CI: 2.10–18.05)] had an increased chance of having three obesogenic behaviors.

## DISCUSSION

The findings of this study indicate that obesogenic behaviors are highly prevalent among university students and tend to co-occur, with more than 80.0% of students presenting at least one obesogenic behavior and 17.9% presenting three behaviors simultaneously. We found a higher chance of one or more obesogenic behaviors in students aged 20 years or younger who self-reported colored skin, self-rated their health as bad, and reported symptoms of depression.

Adopting inadequate eating practices was the single most prevalent risk behavior among university students and was associated with the co-occurrence of two or more obesogenic behaviors. With the transition from high school to higher education, university students face many changes, such as lack of time due to studies, overlapping activities, and new responsibilities, which may interfere with adopting healthy eating practices.^30^ In addition, many factors, such as socioeconomic status, lack of ability to make healthy food choices, difficulty cooking, lack of healthy food in university cafeterias, and “environmental barriers,” such as opening hours of nearby food stores, influence the availability of food, and negatively affect students’ eating behaviors.^30,31^

These factors may favor new eating habits, reflected in unhealthy eating practices and health-related problems, including being overweight.^32-34^ Thus, health promotion strategies, including promoting healthy eating in the university environment, are vital, as numerous health behaviors are developed and established during this period^30^ and tend to continue into adulthood, increasing the risk of developing chronic diseases in subsequent years.^15^

Exposure to health-risk behaviors has been described in studies with young populations.^9^ Studies that evaluated the aggregation of inadequate eating practices and SB showed that these factors share contextual determinants and influence each other.^35^ In the present study, we found that the most prevalent combination of risk behaviors among university students was the coexistence of inadequate eating practices and SB. In contrast, in a study of adults, SB, including the habit of watching television, using a computer, reading books, or magazines, remained associated with the consumption of healthy and unhealthy foods.^36^ However, comparisons between the risk factors analyzed should be interpreted with caution, given the various methods used to assess food intake. It is noteworthy that the instrument used in the present study included other dimensions of adequate and healthy eating and food intake.

Scientific evidence shows that SB reduces energy expenditure and favors inadequate food consumption, including increased intake of foods rich in fat and sugars and decreased consumption of healthy foods such as fruits and vegetables.^35,36^ Moreover, besides being risk factors for becoming overweight, this association between high screen time and inadequate eating habits may increase susceptibility to other health-risk behaviors,^35^ resulting directly in series of unfavorable health outcomes.^37^

The simultaneous occurrence of the three risk behaviors assessed, characterized by inadequate eating practices, leisure-time physical inactivity, and SB, was prevalent in 17.9% of university students. Few studies have investigated the clustering of health risk behaviors among university students.^13^ In a study conducted with Brazilian university students, a high prevalence was observed for the simultaneous occurrence of the four primary behavioral risk factors for NCDs: physical inactivity, inadequate fruit and vegetable consumption, excessive alcohol consumption, and smoking.^38^ The study did not include SB in its analyses since this risk factor has been less studied than other risk behaviors already established in the literature, such as food intake and physical activity. It is worth noting the importance of investigating the aggregation of traditional and emerging risk behaviors among young people, especially university students, to provide information on which to base future actions.^13^

In this study, the co-occurrence of obesogenic behaviors was associated with students’ skin color, differing from the findings of Cureau, Duarte, and Teixeira,^38^ who found no association between the simultaneous presence of three or more behavioral risk factors and skin color of university students. Studies that have evaluated this association are scarce, making comparisons difficult. However, there is evidence showing that ethnic and racial minorities, the black community in particular, have a high prevalence of obesity and obesogenic behavior. Social inequalities make access to health difficult for groups that live in the same environment, such as universities. In this context, studies highlight the urgent need for broad-based, affirmative actions and policies to overcome racial disparities.^39,40^

In this study, we also observed that university students who self-rated their health as bad had a higher chance of one, two, or three obesogenic behaviors than those who self-rated their health as good. Studies show that individuals who perceive their health as bad tend to present more health risk behaviors,^41^ such as inadequate intake of fruits and vegetables, physical inactivity, and SB. These behaviors are determinants of NCDs and are related to the negative subjective assessment of health.^42^ Thus, these findings highlight the importance of considering how health is perceived by university students since perceptions of health can influence the adoption of healthy lifestyle behaviors.^43^

The presence of depressive symptoms was associated with the co-occurrence of obesogenic behaviors among university students, corroborating the findings of Champion et al.,^13^ who evaluated 18-year-old Australian youth and observed a significant association between the clustering of multiple health-risk behaviors and mental health outcomes such as anxiety and depression. One hypothesis to justify this association is that individuals may engage in unhealthy behaviors to help cope with mental health problems.^44^ In addition, stress and mental health disorders may interfere with a person’s choice to adopt healthy lifestyle behaviors such as physical activity, while also exposing themselves to health-risk behaviors.^45^

There was a greater chance of exposure to multiple obesogenic behaviors among university students aged 20 years or younger. Evidence shows that the prevalence of simultaneous exposure to health risk behaviors increases with age,^9,46^ since young people acquire greater autonomy and economic independence with advancing age.^9^ However, this association has not been well established in the literature. In a systematic review of the co-occurrence of multiple risk behaviors, older age groups were considered risk factors for aggregating multiple risk behaviors.^12^

Although the findings of this study are consistent with those reported in the literature, some limitations should be considered when interpreting the results. Students from a single university were included, limiting comparisons with students from other higher education institutions. Another limitation of the study is the methodological design, which does not establish a cause-effect relationship between the variables and temporal relationships on the associations found. In addition, as the students were evaluated in their initial semester at the university, their recent entry into academic life may not have set their lifestyles.

It is important to highlight that this study was based on self-reported behaviors, which may have generated information bias, as young people tend to overestimate or underestimate their exposure to health risk behaviors. Despite these limitations, the findings obtained add essential evidence regarding the prevalence and factors associated with the co-occurrence of obesogenic behaviors among university students.

## CONCLUSIONS

The findings from this study showed that a high proportion of university students with simultaneous obesogenic behaviors, especially among those who self-reported colored skin, rated their health as bad, and reported depressive symptoms. These findings contribute to a better understanding of the associations between various obesogenic behaviors, highlighting the need for interventions directed at university students. In addition, these results highlight the importance of health promotion in the university environment, with actions aimed at a healthy lifestyle. Public policies that target risk behaviors in groups and stimulate a healthy food environment and physical activity in universities are essential for reducing the risk of major chronic diseases related to excess weight.
